# Supplementary material for: The patterns of alternative TSS usage explain the highly heterogeneous landscape of 5′UTR lengths in eukaryotes
Source: NAR Genom Bioinform. 2025 Nov 11;7(4):lqaf152. doi: 10.1093/nargab/lqaf152 (PMC12604664; doi:10.1093/nargab/lqaf152)
Supplement: lqaf152_Supplemental_Files [file lqaf152_supplemental_files.zip › Supplementary_Tables_Figs_0907.pdf]

Table S1. Mapping statistics of CAGE datasets in yeast species

sample	total	overall alignment rate	unique mapped	unique mapped rate
ScerAr.1	39938546	94.93%	27771775	69.54%
ScerAr.2	44730229	94.75%	31569089	70.58%
ScerDD.1	29567980	95.04%	14434829	48.82%
ScerDD.2	24919356	95.42%	11560704	46.39%
ScerDS.1	35161190	95.66%	22129261	62.94%
ScerDS.2	36682729	95.60%	22315465	60.83%
ScerGa.1	45714017	95.40%	22366435	48.93%
ScerGa.2	39070001	94.80%	20978144	53.69%
ScerGl.1	42056614	95.36%	21851098	51.96%
ScerGl.2	38553431	95.18%	20938226	54.31%
ScerH2.1	41669661	95.41%	18871366	45.29%
ScerH2.2	17907214	95.22%	8425339	47.05%
ScerHS.1	43747268	95.67%	19614393	44.84%
ScerHS.2	25762666	95.73%	9090311	35.28%
ScerNa.1	34638352	95.11%	19977331	57.67%
ScerNa.2	19037627	94.72%	10363229	54.44%
ScerYP.1	29740675	95.17%	17346210	58.32%
ScerYP.2	30005902	95.88%	20196591	67.31%
Spar.1	41471738	89.26%	21260671	51.27%
Spar.2	43638383	83.48%	20380879	46.70%
S. castellii-1	36763702	96.25%	29365574	79.88%
S. castellii-2	37560080	96.06%	29540910	78.65%
Sch. pombe -1	41068801	90.87%	32673739	79.56%
Sch. pombe -2	35638178	90.11%	28826177	80.89%

Table S2.A list of omics data used in this study

Dataset	Species	Strain/Cell line	Condition	Growth phase	Temperature	CHX treatment	rRNA deletion	Type
PRJNA483730	S. cerevisiae	BY4741	Ar: Arrest, α factor (2.5 mM) for 45 min; add another 50 μL to 25 mL yeast for another 30 min	log-phase	30°C	N.A	N.A	CAGE
PRJNA483730	S. cerevisiae	BY4741	YP: YPD	log-phase	30°C	N.A	N.A	CAGE
PRJNA483730	S. cerevisiae	BY4741	DD: 1 mM MMS for 1 h	log-phase	30°C	N.A	N.A	CAGE
PRJNA483730	S. cerevisiae	BY4741	DS: DSA, YPD medium for 48 h	log-phase	30°C	N.A	N.A	CAGE
PRJNA483730	S. cerevisiae	BY4741	Ga: YP medium with 2% galactose	log-phase	30°C	N.A	N.A	CAGE
PRJNA483730	S. cerevisiae	BY4741	Gl: YP medium with 16% glucose	log-phase	30°C	N.A	N.A	CAGE
PRJNA483730	S. cerevisiae	BY4741	H2: Add H_2O_2 to early-log phase cells for a final concentration of 0.20 mM, 30 min	log-phase	30°C	N.A	N.A	CAGE
PRJNA483730	S. cerevisiae	BY4741	HS: Heat shock from 30°C to 37°C, 1 h	log-phase	30°C	N.A	N.A	CAGE
PRJNA483730	S. cerevisiae	BY4741	Na: Add NaCl for a final concentration of 1 M for 45 min	log-phase	30°C	N.A	N.A	CAGE
PRJNA510689	S. paradoxus	N17	YPD	log-phase	30°C	N.A	N.A	CAGE
PRJNA510689	Sch. pombe	JB1171	YPD	log-phase	30°C	N.A	N.A	CAGE
FONTOM5	Homo sapiens	K562	RNA directly extracted from stock	N.A	37°C	N.A	N.A	CAGE
FONTOM5	Homo sapiens	HepG2	RNA directly extracted from stock	N.A	37°C	N.A	N.A	CAGE
PRJNA313018	Mus musculus	NIH3T3	DMEM	N.A	37°C	N.A	N.A	CAGE
PRJNA313018	Mus musculus	NIH3T3	DMEM	N.A	37°C	Yes	no	Polysome profiling
SRP187756	S. cerevisiae	S288C	H2: Add H_2O_2 to early-log phase cells for a final concentration of 1.5 mM, 30 min	log-phase	30°C	100 μg/ml	N.A	Polysome profiling
SRP187756	S. cerevisiae	S288C	YP: YPD	log-phase	30°C	100 μg/ml	N.A	Polysome profiling

Table S3. Statistics of ATU and DE genes in different conditions comparing to YPD control

Condition	# Gene with ATU	#gene without ATU	# Gene with ATU and DE	# Non ATU Gene with DE	#total
Cell Arrest	142	4839	77	628	4981
DNA Damage	108	4720	56	1254	4828
Diauxic Shift	191	4644	145	2269	4835
Galactose	145	4966	93	1935	5111
Glucose	130	4711	75	1090	4841
H_2O_2	109	4863	57	746	4972
Heat Shock	188	4615	141	1774	4803
Nacl	144	4905	70	1469	5049

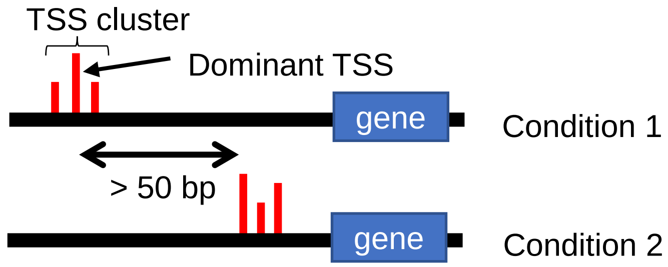

Supplementary Figure S1. An illustrative depiction outlining the definition of ATU. Firstly, the distribution of the two TSS clusters is statistically different as determined through the utilization of a paired Wilcoxon Test ($p < 0.05$); Secondly, the dominant TSS in each TSS cluster displays a certain distance (≥ 50 bp).

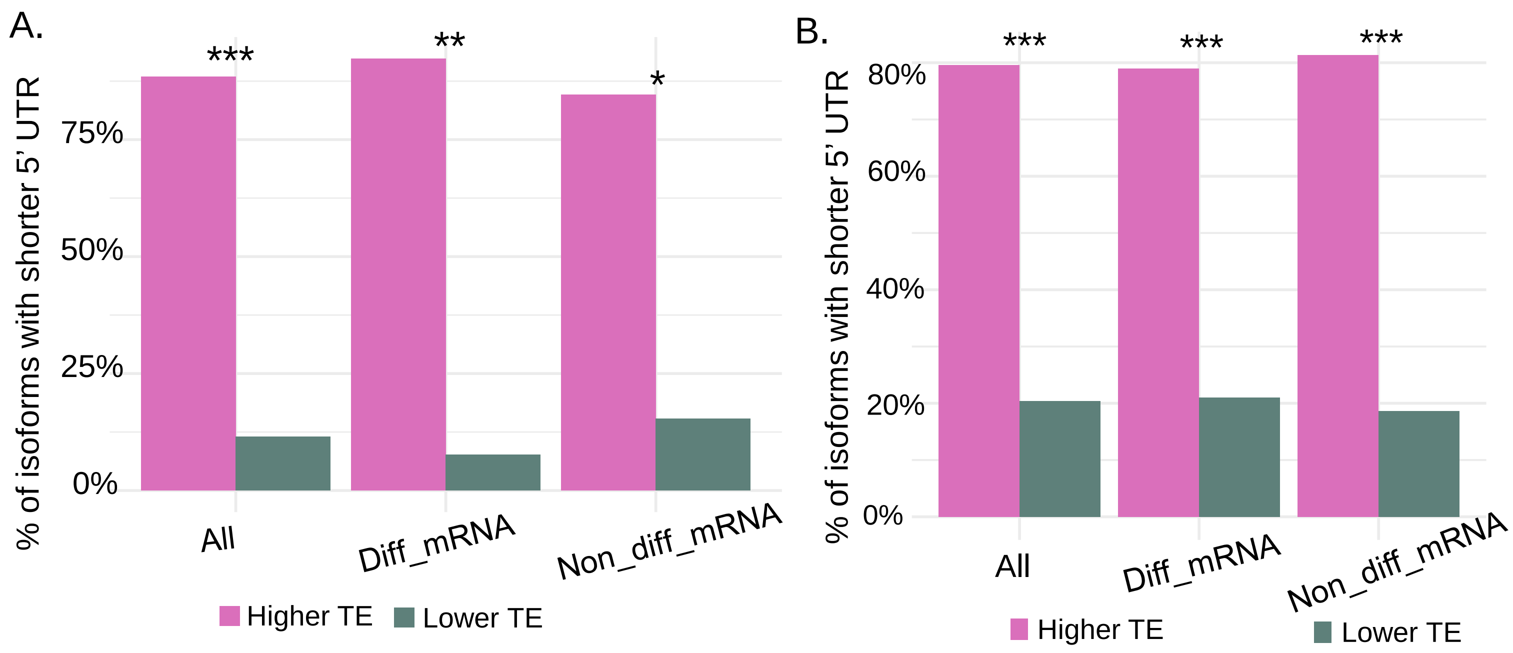

Supplementary Figure S2. Gene with long 5'UTR tend to have a low TE and the effect is independent of mRNA level (A), Diff_mRNA" refers to the group of genes that there is significant difference in the abundance between mRNA isoforms (|mRNA2/mRNA1| ≥ 2). "Non_diff_mRNA" refers to the groups of genes which there is no significant difference in the abundance between mRNA isoforms (|mRNA2/mRNA1| < 2). Only genes with significant TE are compared (|TE2/ TE 1| ≥ 1.5), the data was from *S. cerevisiae*. **(B)** similar to (A) except for the data was from mouse fibroblasts (NIH3T3). *: $p < 0.05$, **: $p < 0.01$, ***: $p < 0.001$, binomial test.

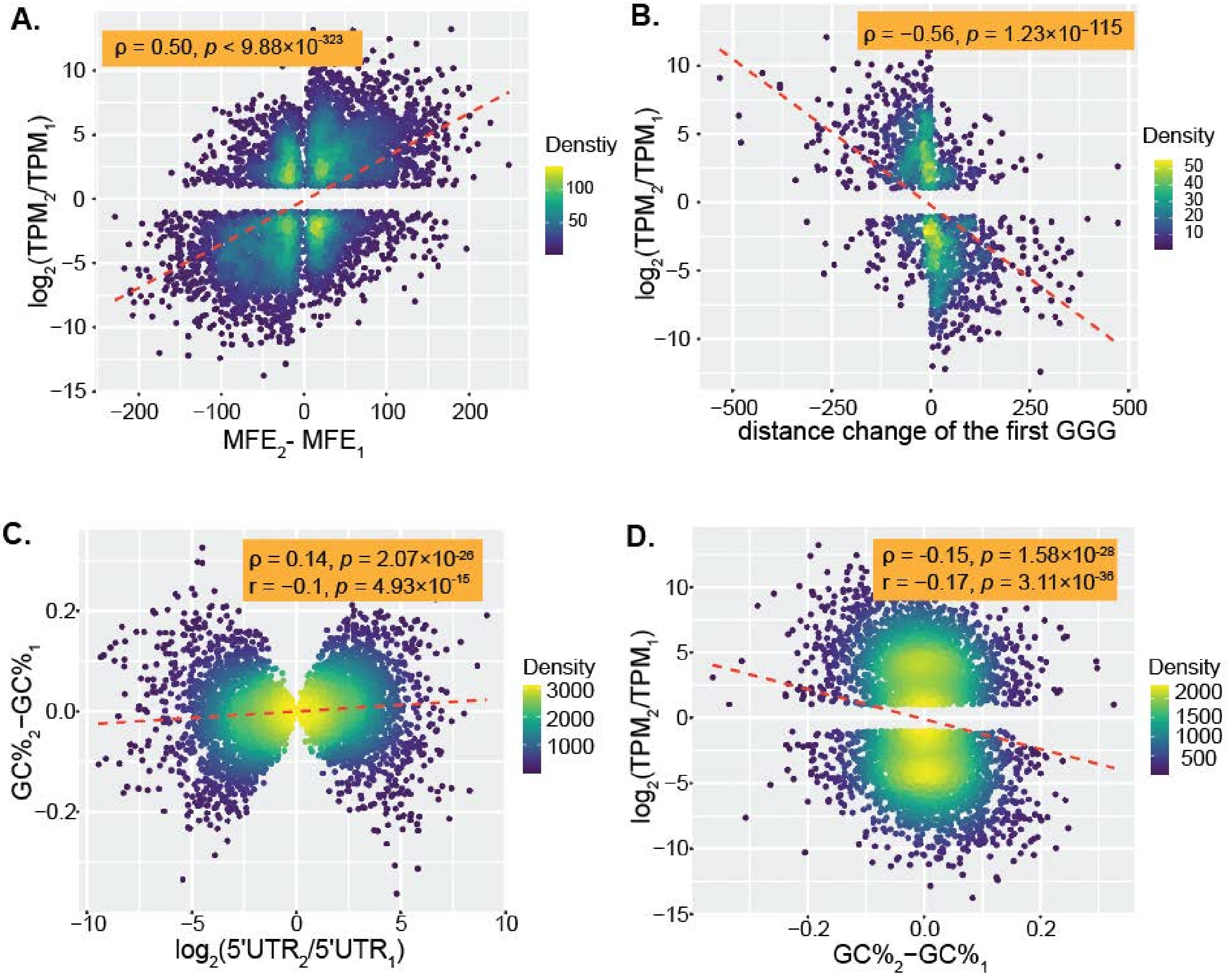

Supplementary Figure S3. Correlation between different motif characteristic and peak TPM or changes of peak TPM ($\log_2$). (A) A positive correlation between changes of MFE [x = MFE_2 - MFE_1] and fold changes of transcript abundance (y = $\log_2(TPM_2/TPM_1)$.
(B) A negative correlation between change of the distance of the first GGG to TSS (dGGG2-dGGG1) and fold changes of transcript abundance (y = $\log_2(TPM_2/TPM_1)$ from transcription isoforms generated by the same gene. Each dot represents a pair of 5'UTR isoforms in S. cerevisiae.**(C)** Scatter plot showing a positive correlation between $\log_2$ ratios of 5'UTR [x = $\log_2(5'UTR_2/5'UTR_1)$] and changes of GC% content [y=$GC\%_2$-$GC\%_1$] from transcription isoforms generated by the same gene. **(D)** Scatter plot showing a negative correlation between changes of GC% content [x=$GC\%_2$-$GC\%_1$] and peak TPM values [y = $\log_2(TPM_2/TPM_1)$] from transcription isoforms generated by the same gene.

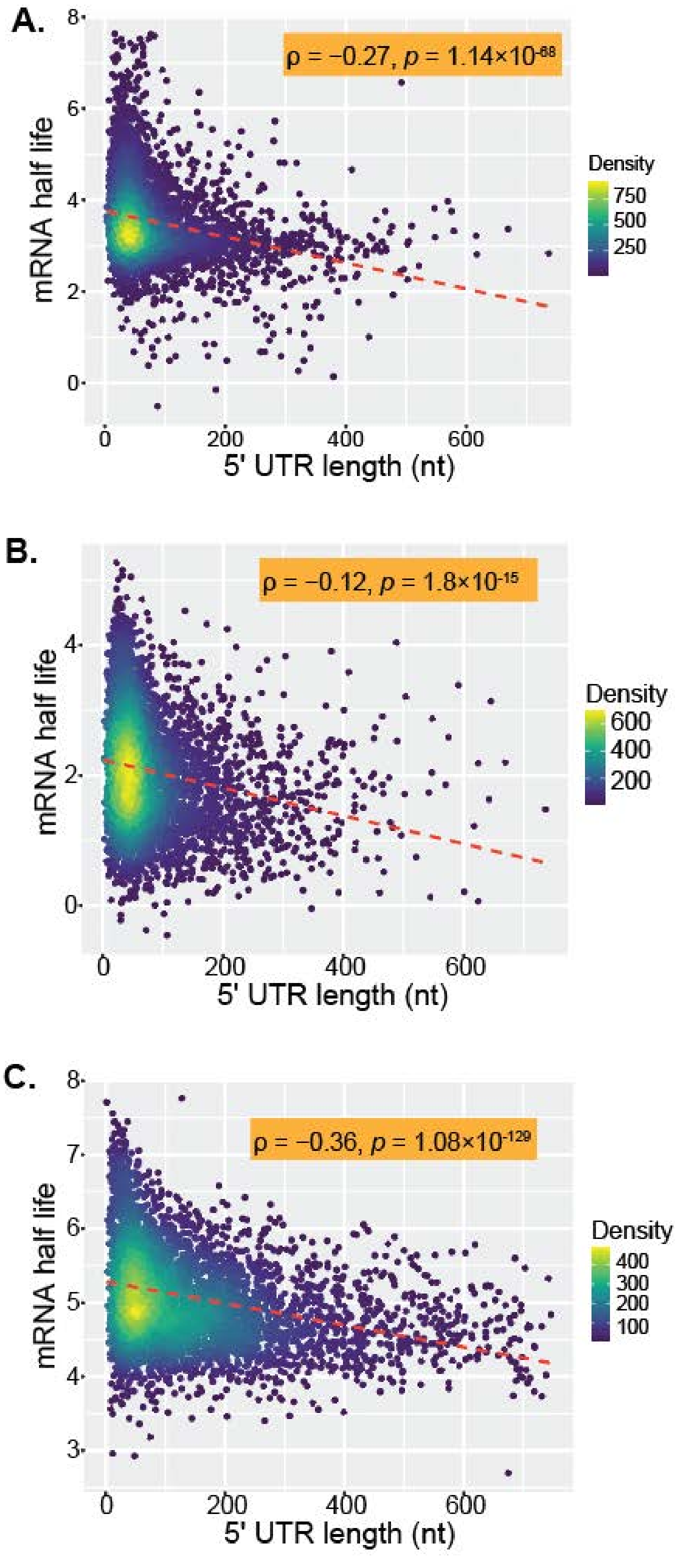

Supplementary Figure S4. 5'UTR lengths is anticorrelated with mRNA half life.
(A) Negative correlations between 5'UTR lengths and mRNA half life. X axis is the weighted 5' UTR length and Y axis is the mRNA half life data from Gresham et al 2015. **(B)** Negative correlations between 5'UTR lengths and mRNA half life in *S. cerevisiae*. X axis is the weighted 5' UTR length and Y axis is the mRNA half life data. The mRNA half data is from Chan et al 2018. **(C)** Negative correlations between 5'UTR lengths and mRNA half life in *Sch. pombe*. The mRNA half data is from Mata et al 2015.

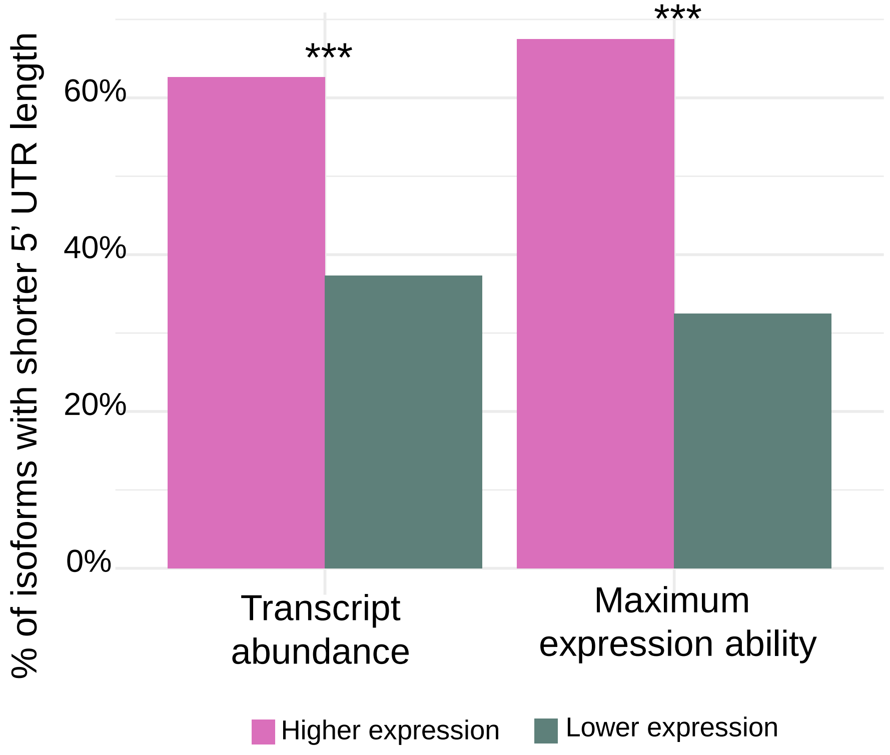

Supplementary Figure S5. Transcript with short 5’ UTR length tend to have high expression levels Maximum expression ability: Peak expression of transcript isoform in nine conditions. ***: $p < 0.001$, binomial test. Only transcript isoform pairs with significant different expression are compared ($|mRNA_2/ mRNA_1| \geq 2$).

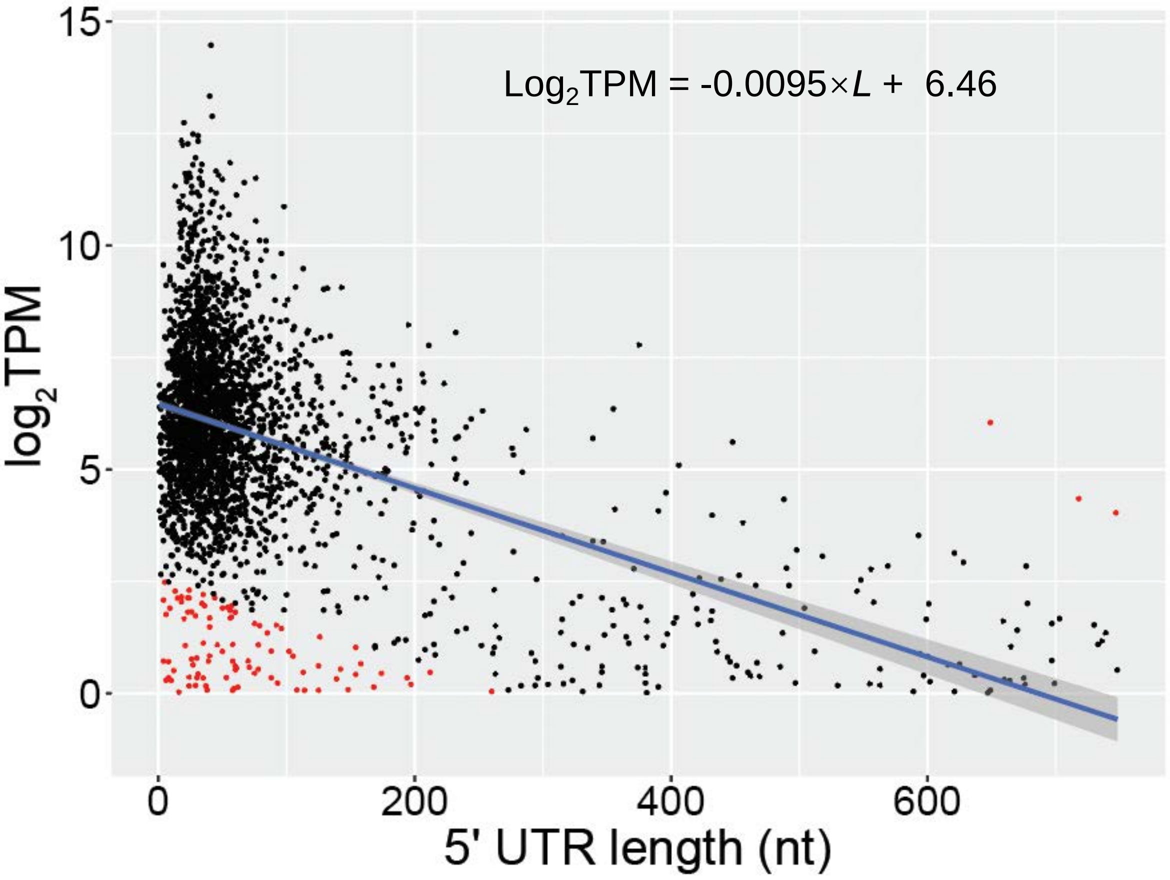

Supplementary Figure S6. A simple linear model shows the relationship between 5' UTR lengths and peak expression level from gene with a single core promoter. Each dot represent a gene and dots in red color were identified as genes with a non-optimal 5'UTR length or outlier genes.

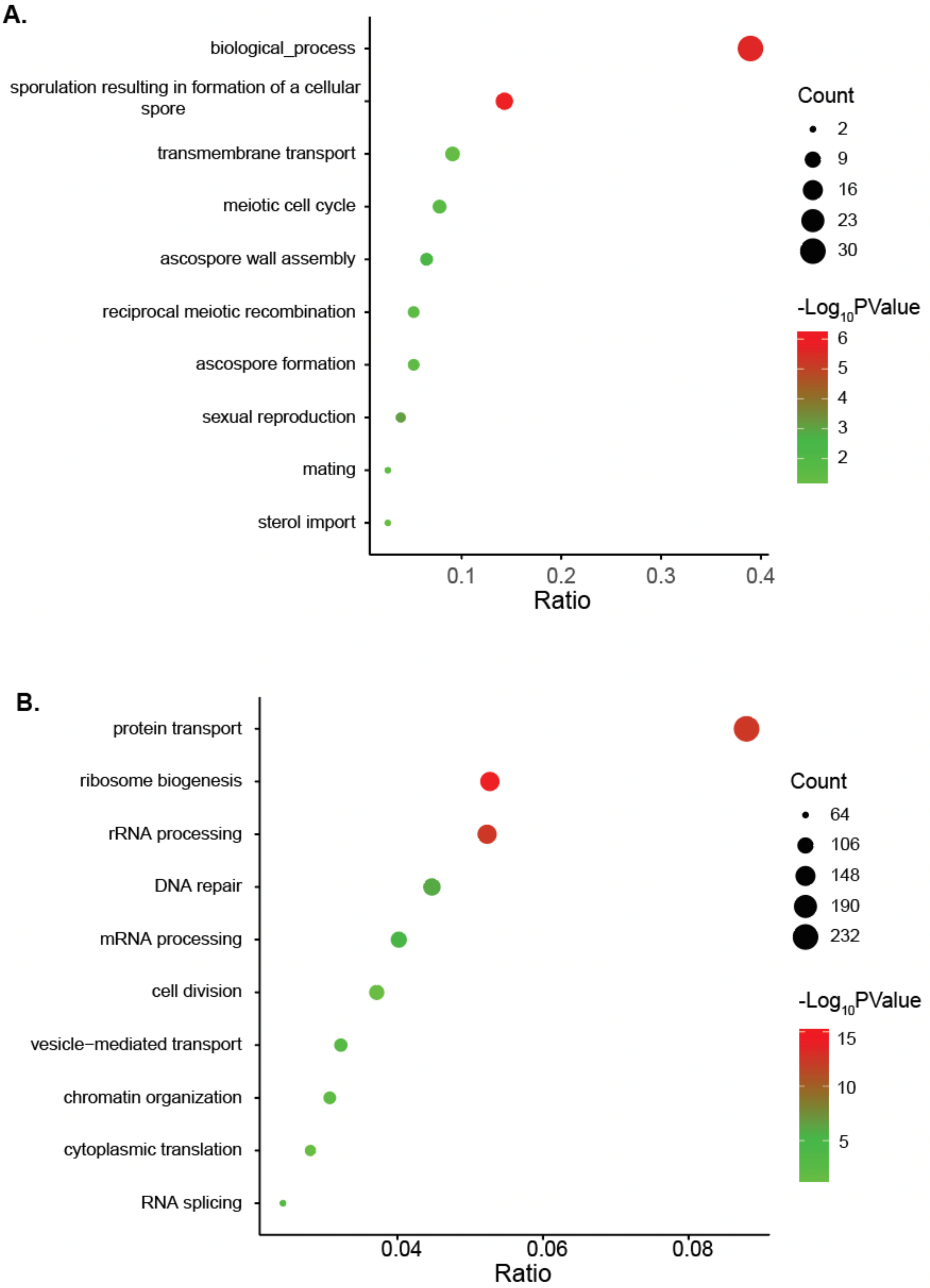

Supplementary Figure S7. Functional enrichment of 5'UTR optimal and non-optimal genes. (A) KEGG pathway enrichment of 5'UTR non-optimal genes **(B)** KEGG pathway enrichment of 5'UTR optimal genes**.**

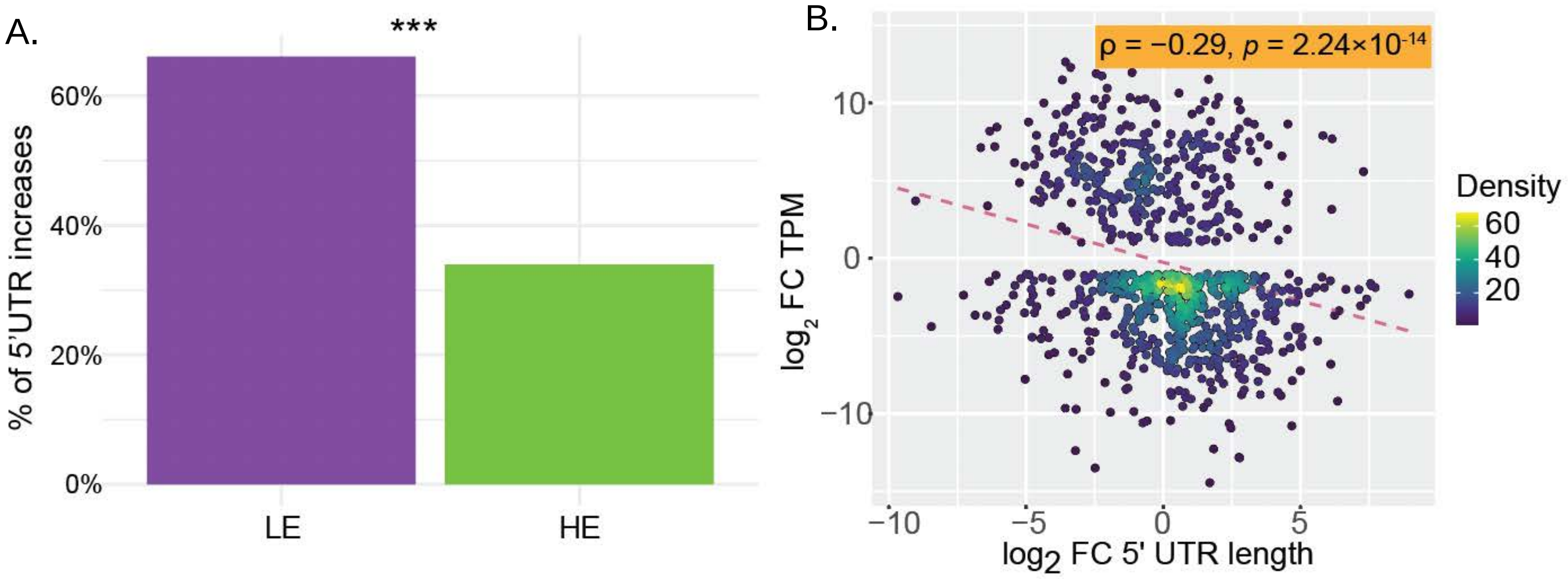

Supplementary Figure S8. Orthologous gene pairs with lower expression (LE) tend to have longer 5′UTRs using human cell line data. (A) % of transcripts 5'UTR increase when mRNA expression decrease (LE) or increases (HE). ***: $p < 0.001$. The *p* value is from a binomial test of the null hypothesis of equal numbers of genes in the two groups. **(B)** FC of 5' UTR length anticorrelates with FC of gene expression by comparing TSS maps of two human cells lines (HepG2 and K562); The X-axis shows FC of 5' UTR length ($\log_2$), the Y-axis show FC of gene expression ($\log_2$). The genes with significant changes of transcription level (FC ≥2) and ATU (p <0.05, wilcox.test) were identified between two cell lines.

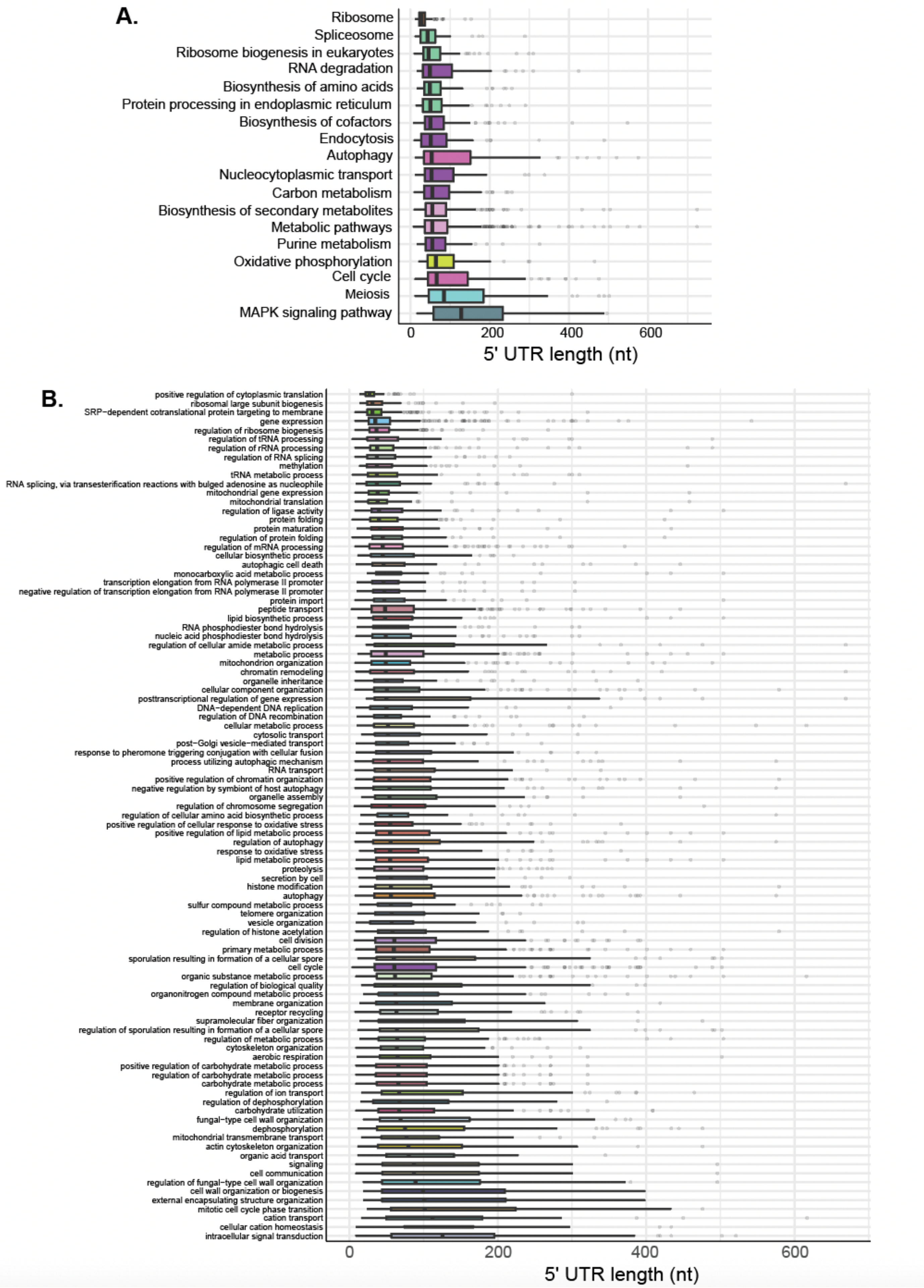

Supplementary Figure S9. Distribution of 5’UTR length among different types of genes. (A) Box plots showing large variations in 5′ UTR lengths among genes from different KEGG pathways. **(B)** Box plots showing large variations in 5′ UTR lengths among genes from different GO terms.

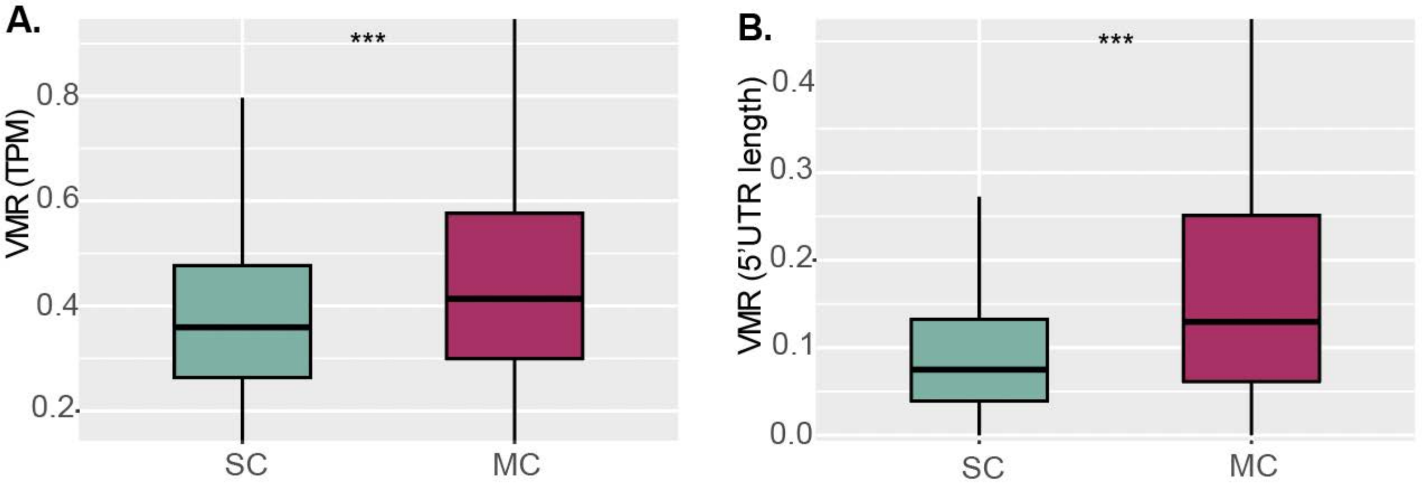

Supplementary Figure S10. Higher variance of gene expression and 5'UTR length in MC genes than SG genes. **(A)** Boxplot showing a much higher relative expression variance in MC genes than SC genes in *S. cerevisiae*. The relative variance, or variance-to-mean ratio (VMR) was calculated as the standard deviation of TPM values across nine growth conditions normalized by its mean. **(B)** Boxplot showing a much higher relative variance of 5' UTR length in MC (multiple-cluster) genes than SC (single-cluster) genes. The relative variance was calculated as the standard deviation of 5'UTR length values across nine growth conditions normalized by its mean. ***: $p < 0.001$, Student's t-test.
